# Cerebrospinal fluid penetration of targeted therapeutics in pediatric brain tumor patients

**DOI:** 10.1186/s40478-020-00953-2

**Published:** 2020-06-03

**Authors:** Armin Sebastian Guntner, Andreas Peyrl, Lisa Mayr, Bernhard Englinger, Walter Berger, Irene Slavc, Wolfgang Buchberger, Johannes Gojo

**Affiliations:** 1 Institute of Analytical Chemistry, Johannes Kepler University, Linz, Austria; 2 Department of Pediatrics and Adolescent Medicine and Comprehensive Center for Pediatrics, Medical University of Vienna, Währinger Gürtel 18-20, 1090 Vienna, Austria; 3 Institute of Cancer Research, Department of Medicine I, Medical University of Vienna, Vienna, Austria

**Keywords:** Blood-brain barrier, Cerebrospinal fluid, Pharmacokinetics, Ommaya reservoir, High performances liquid chromatography mass spectrometry, Targeted therapy, Precision medicine

## Abstract

Treatment with small-molecule inhibitors, guided by precision medicine has improved patient outcomes in multiple cancer types. However, these compounds are often not effective against central nervous system (CNS) tumors. The failure of precision medicine approaches for CNS tumors is frequently attributed to the inability of these compounds to cross the blood-brain barrier (BBB), which impedes intratumoral target engagement. This is complicated by the fact that information on CNS penetration in CNS-tumor patients is still very limited. Herein, we evaluated cerebrospinal fluid (CSF) drug penetration, a well-established surrogate for CNS-penetration, in pediatric brain tumor patients. We analyzed 7 different oral anti-cancer drugs and their metabolites by high performance liquid chromatography mass spectrometry (HPLC-MS) in 42 CSF samples obtained via Ommaya reservoirs of 9 different patients. Moreover, we related the resulting data to commonly applied predictors of BBB-penetration including ABCB1 substrate-character, physicochemical properties and in silico algorithms. First, the measured CSF drug concentrations depicted good intra- and interpatient precision. Interestingly, ribociclib, vorinostat and imatinib showed high (> 10 nM), regorafenib and dasatinib moderate (1–10 nM) penetrance. In contrast, panobinostat und nintedanib were not detected. In addition, we identified active metabolites of imatinib and ribociclib. Comparison to well-established BBB-penetrance predictors confirmed low molecular weight, high proportion of free-drug and low ABCB1-mediated efflux as central factors. However, evaluation of diverse in silico algorithms showed poor correlation within our dataset. In summary, our study proves the feasibility of measuring CSF concentration via Ommaya reservoirs thus setting the ground for utilization of this method in future clinical trials. Moreover, we demonstrate CNS presence of certain small-molecule inhibitors and even active metabolites in CSF of CNS-tumor patients and provide a potential guidance for physicochemical and biological factors favoring CNS-penetration.

## Introduction

Brain tumors are the most frequent solid tumors in childhood and the leading cause of cancer-related death in this age group [1]. This fact can be attributed to several factors including the particular aggressiveness of certain tumor types, but also to the lack of effective therapeutic strategies for relapsed patients [2, 3]. Continuous effort of both academia and pharmaceutical companies has resulted in the identification of multiple promising therapeutic targets as well as targeted inhibitors for the treatment of brain tumors, which can be detected by precision medicine approaches [4, 5]. As a consequence effective targeted treatment approaches such as BRAF- [6] and NTRK-inhibitors [7] are already applied in the treatment of brain tumors. However, for the majority of newly identified targets the implementation of preclinical findings into routine clinical application based on successful clinical trials is limited [4].

This gap is widely attributed to the fact that penetrance of anti-cancer drugs to the central nervous system (CNS) is limited by the blood-brain barrier (BBB) and blood-CSF-barrier, which prevent potentially effective drugs from engaging their targets within the tumor tissue [8]. The BBB represents a unique and complex structure at the capillaries within the CNS. It is composed of various different cell types including endothelial cells, pericytes and neural cells, each playing a distinct role in the maintenance of the BBB. The central element of this barrier are the endothelial cells, which are joined together by tight junctions preventing most drugs from passively diffusing into brain parenchyma [8, 9]. Moreover, endothelial cells express efflux pumps including ABCB1 and ABCG2 which actively export molecules to the luminal surface and thus into the blood stream [8, 10, 11]. The integrity of the BBB is altered by pathogenic events such as tumorigenesis [8, 12]. This is demonstrated by penetration of compounds with low molecular weight (MW) such as gadolinium, which is used as contrast agent in magnetic resonance imaging (MRI) examinations, into the tissue of certain tumors,. However, multiple studies suggest that the BBB is more or less intact in the majority of brain tumors [9]. In addition, it has long been known that treatment of childhood brain tumors with cerebrospinal fluid (CSF) dissemination is limited by the relative inaccessibility of CSF to systemically administered drugs not crossing the BBB. This corroborates the notion that brain tumors are also protected from anti-cancer drug exposure via components of the BBB [13–16].

Based on the above-mentioned facts, it is indispensable for an effective anti-brain-tumor treatment that the respective remedies cross the BBB. Consequently, it is generally considered that preclinical as well as pharmacokinetic assessment of novel small molecule inhibitors against brain tumors should include evaluation of BBB penetration [4, 17]. As an example, cell accumulation and efflux competition assays enable prediction whether a molecule is a substrate/inhibitor of either ABCB1 or ABCG2, which will most likely limit penetration into the brain tissue [18]. Apart from these biological assessments, additional in silico tools have been developed to predict CNS penetration based on chemical structure, taking into account the physicochemical properties of the respective compounds. These properties generally include lipophilicity, polar surface area (PSA), the number of hydrogen-bond donors (HBD) and acceptors (HBA), the number of rotatable bonds, the charge at the given pH, and the MW [17, 19, 20]. Recently, we described collision cross sections (CCS), which characterizes molecular volume and molecular branching as an additional reliable predictor for CNS penetration [21].

Besides, plasma protein binding is a key parameter for sufficient CNS penetration [22–24]. Protein binding of drugs is generally related to albumin binding, but is in fact more complex, as various other proteins such as glycoproteins, globulins, and lipoproteins may also play a role [25, 26]. Since the free level of a drug (the fraction which is not bound to matrix proteins) within a biological fluid may represent the amount of a substance actually exhibiting pharmacologic effects, it is crucial to distinguish the measured total drug levels from free drug levels [4, 17, 27]. This is generally referred to as free drug hypothesis. Recent studies have shown the importance of the determination of free levels for CNS drugs [4, 17, 25, 27, 28]. However, the analytical access to free levels is generally associated with considerable expenditure on equipment [29, 30], especially in CSF, where the total concentrations of drugs are significantly lower compared to serum [25, 31]. As protein levels in CSF are multiple orders of magnitude lower than in serum, protein binding is therefore usually neglected in CSF. CSF and brain parenchyma are only separated by a thin permeable ependymal tissue layer and ventricular CSF very well reflects the unbound drug amount. Consequently, drug concentrations in CSF are generally considered as valid surrogate for concentrations in the extracellular CNS fluid [32–35].

In contrast to the identification of multiple tumor targets across pediatric brain tumors for precision medicine-guided treatment [3], information on CNS penetration of small molecule inhibitors in pediatric CNS tumor patients is generally limited. To overcome this lack of translational validation of BBB penetration, in the present study we analyzed concentrations of seven different orally administered small molecule inhibitors in the CSF of pediatric brain tumor patients. In parallel, we comprehensively compared established in vitro and in silico methods to predict CNS-penetration to our real-world dataset in order to provide information on the validity of the different prediction approaches.

## Materials and methods

### Patients, sample material and instrumental analysis

Samples were obtained from patients treated at the Department of Pediatrics and Adolescent Medicine at the General Hospital of Vienna and the study was approved by the ethics committee of the Medical University of Vienna. Patient characteristics (age, weight, sex) and clinical data (tumor type, medication, radiotherapy, localization, leptomeningeal disease, serum protein levels, and CSF protein levels) were retrieved from patient charts. Clinical and serum parameters were measured within the same treatment cycle, CSF protein levels at the same date and sample collection time point. Samples were collected and snap frozen within routine clinical sampling during intraventricular chemotherapy administration.

**Fig. 1:**
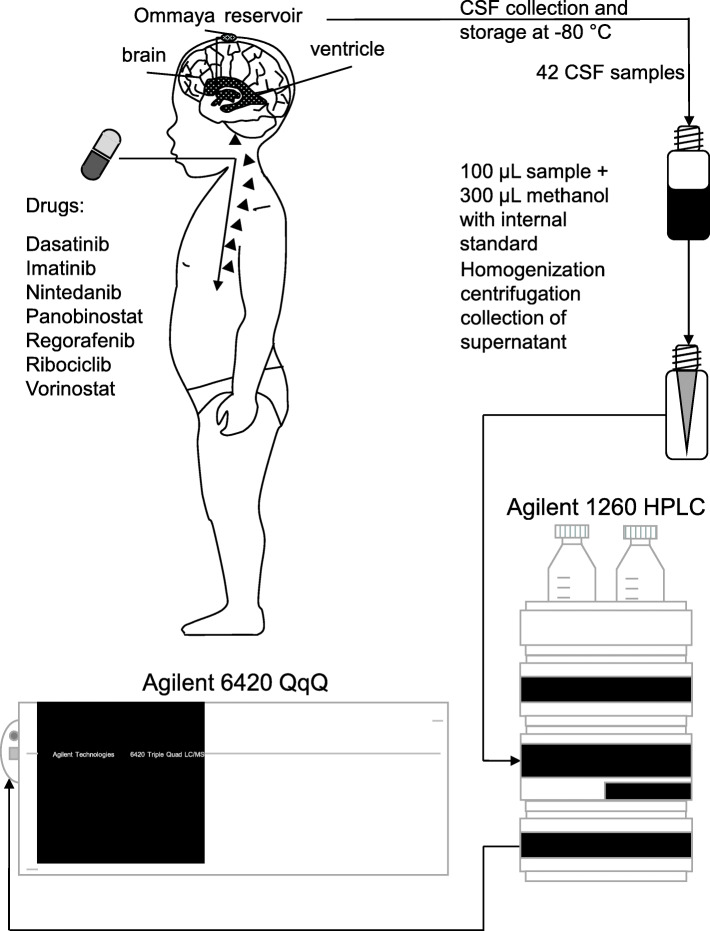
Schematic depiction of the study workflow. Cerebrospinal fluid (CSF) was collected at steady state from pediatric brain tumor patients receiving oral small molecule inhibitors. CSF samples were analyzed using protein precipitation prior to HPLC-QqQ MS analysis

We included samples of patients who received oral small molecule inhibitors and concomitant intraventricular therapy. The investigated substances included dasatinib (1 patient, 3 samples), imatinib (3 patients, 12 samples), nintedanib (3 patients, 11 samples), panobinostat (1 patient, 2 samples), regorafenib (1 patient, 3 samples), ribociclib (1 patient, 3 samples), and vorinostat (2 patients, 9 samples). As backbone therapy the majority of patients were treated according to the MEMMAT protocol (NCT01356290) [36] with slight modifications in some cases as indicated in Additional file 1: Table S1. Dosing for pediatric patients was adjusted according to the treatment protocols or previous reports as outlined in Table 1. Interpatient differences in dosing per body weight may result from individual drug tolerability and adapted dosing in each case, as well as the limitation that for none of the investigated drugs oral solutions for body weight adjusted dosing were available, making it necessary to use drug formulations standardized for use in adult patients. In two cases, samples of patients being sequentially treated with different drugs were collected as indicated in Additional file 1: Table S2. In general, we aimed at including multiple samples over the whole treatment period after reaching steady state (Additional file 1: Table S1, range 1–31 weeks). The earliest time point for sample analysis was defined as > 5 times the half-life if not otherwise indicated. Consequently, the range of the earliest analyzed sample was from 3 days for vorinostat (half-life 2 h [37]) to 9 days for panobinostat (half-life 31 h [38]). In addition, one matched serum sample for nintedanib (taken simultaneously to one CSF sample to prove oral uptake) was available for analysis. An overview of patient characteristics and samples is provided in Table 1. Leptomeningeal disease was present in all but one patient receiving nintedanib. CSF samples (< 0.5 mL) of pediatric patients were collected using an Ommaya reservoir, immediately snap frozen and afterwards stored at − 80 °C prior to HPLC-MS analysis. Figure 1 gives general information on the workflow of CSF sample collection, sample preparation and HPLC-MS analysis. Detailed information on the analysis method and the working parameters of the HPLC-MS system(s) can be found in the supplementary material available online (Additional file 1: Figures S1 and S2 and Tables S3–4).

**Table 1:** Overview of the pediatric tumor patients with detailed information on age, sex, radiotherapy, histology, localization, metastasis and dosage

Drug	Patient #	Age	Sex	RTX	Tumor histology	Localization	Lepto-meningeal metastasis	Dose mg/kg
Imatinib	1	18,3	m	focal	Germ cell tumor	pineal	yes	8.51 BID
	2	14.5	f	focal	Glioblastoma	hemispheric	yes	1.82 QD
	3	7.5	m	CSI	Plexuscarcinoma	hemispheric	yes	16.67 QD
Dasatinib	1	19.1	m	focal	Germ cell tumor	pineal	yes	1.72 BID
Nintedanib	4	19.4	m	focal	Ependymoma	hemispheric	yes	3.72 BID
	5	13.5	m	focal	Ependymoma	hemispheric	no	3.23 BID
	6	7.6	m	focal	Ependymoma	posterior fossa	yes	10.35 QD
Panobinostat	4	20.3	m	focal	Ependymoma	hemispheric	yes	0.35 3 doses / week
Regorafenib	4	20.1	m	focal	Ependymoma	hemispheric	yes	0.78 QD
Ribociclib	7	8.4	f	focal	Epithelioid sarcoma (metastasis)	non CNS primary	yes	3.33 QD
Vorinostat	8	10.8	m	CSI	Medulloblastoma	posterior fossa	yes	3.74 QD
	9	12.7	f	CSI	Atypical teratoid rhabdoid tumor	hemispheric	yes	2.63 QD

RTX, radiotherapy; CSI, craniospinal irradiation; CNS, central nervous system

### In silico assisted workflow for the identification of metabolites in biological samples and prediction of BBB penetration

Within the scope of this work, in silico based metabolite prediction was performed using ADMET Predictor 9.0 (Simulations Plus, Lancaster, Canada), Pallas 3.8 (CompuDrug International Inc., Bal Harbor, Florida), and Smartcyp 2.4.2 [39–43], revealing mostly phase I metabolites such as oxidation, hydroxylation and hydrolysis products of the tested substances. In a next step, theoretical molecular formulas and related ion masses of the predicted metabolites were transferred into a database and compared to measured high-resolution Q-TOF mass spectra of biological samples. For that purpose, MassHunter Qualitative Workflows B.08.00 software (Agilent Technologies, Santa Clara, California) was used, as it enables (semi)-automated data processing. Within this procedure, theoretical and measured exact ion masses and abundances of isotopologues and molecule-cation adducts were compared. The results of this comparison were then used to evaluate the validity of the identification, which is expressed as a score. This quality measure is important as it reduces the risk of false positives that are likely in complex biological samples, especially when only the accurate mass of a molecule is used.

In addition, ADMET Predictor 9.0 was utilized to predict brain exposition via its integrated algorithm that binary (high or low) estimates BBB penetration and quantitatively predicts the logarithm of the brain to blood partition coefficient. As complementary approach we used the SwissADME predictor [44], which generates a datasheet indicating whether the substance is likely to cross the BBB barrier or not (yes or no).

### Experiments targeting the ABCB1 substrate character of the examined drugs

We first compared interaction of the investigated drugs with ABCB1 by measuring fluorescent substrate drug accumulation in the parental KB-3-1 (a HeLa cell derivative) cell model and its isogenic ABCB1-overexpressing KB-C1 cell model, as previously published [45]. As a known fluorescent ABCB1 substrate calcein (derived from non-fluorescent calcein AM) was used in the presence of investigated drugs (1 μM and 10 μM) using fluorescence-activated cell sorting (FACS). The ABCB1 inhibitor elacridar was used as positive control. As this approach cannot distinguish competitive (by a substrate) and non-competitive (by a pure inhibitor) ABCB1 inhibition, KB-C1 cells were additionally exposed to the examined drugs with or without elacridar to test the actual impact of ABCB1 activity on the concentration of drug within the cells. In detail, cultivated cells were grown, washed, lysed using 100 μL methanol, centrifuged and stored at − 80 C prior to analysis. After thawing, the samples were homogenized, sonicated (15 min, 50 Hz, 0 °C), homogenized again and centrifuged. Finally, 100 μL supernatant were collected and analyzed using the developed HPLC-QqQ MS method (see Supplementary Material).

### Calculation of free drug levels

Directly measuring free drug levels in CSF may be impossible due to low drug concentrations. Besides the experimental quantitation, if feasible, also the law of mass action can be used to predict protein-unbound drug levels [46]. Accordingly, CSF proteins and associated drugs may be considered as center and ligand, forming a complex. Consequently, we applied the corresponding mathematical model to estimate free drug concentration. The derivation is given in further detail in the supplementary material.

## Results

### Feasibility of drug and metabolite detection in CSF derived via Ommaya reservoirs

In a first step, we determined whether CSF samples from Ommaya reservoirs analyzed with HPLC-QqQ MS or HPLC-IMS-Q-TOF MS allow reliable detection of orally administered drugs and the respective metabolites. For that purpose, special focus was on the improvement of an HPLC-QqQ MS multi-method to quantitate the respective substances in CSF samples. The applied chromatographic gradient as well as the sensitivity of the MS detection were optimized to allow a quantitation of all mentioned analytes within one run and over a broad range of analyte concentration levels (800 ng L^− 1^ to 250 μg L^− 1^).

For each analyte, the recovery of the method was tested, including sample preparation and HPLC-QqQ MS analysis, and matrix-matched calibration was used to compensate possible matrix-induced electrospray ionization suppression/enhancement effects, thereby assuring the accuracy of the quantitation. Intra-day and inter-day precision were determined to be ≤8.6% relative standard deviation for all analytes on the basis of five consecutive measurements of artificial samples containing 5 μg L^− 1^ of analyte. Additionally, the developed HPLC-QqQ MS method showed good linearity and an R^2^ ≥ 0.99 in a range between the lower limit of detection and 250 μg L^− 1^. The lower limit of detection (LOD) of the method was determined to be well below 1 μg L^− 1^ for all analytes, (see Supplementary Material). The determination of CCS was performed by the means of HPLC-IMS-Q-TOF MS showing highly reproducible results with intra-day precisions ≤0.1% relative standard deviation (RSD) of 5 consecutive measurements and inter-day precisions ≤0.2% RSD for all analytes, (see Supplementary Material).

### Quantitation of pharmaceuticals including free drug levels and metabolites

**Fig. 2:**
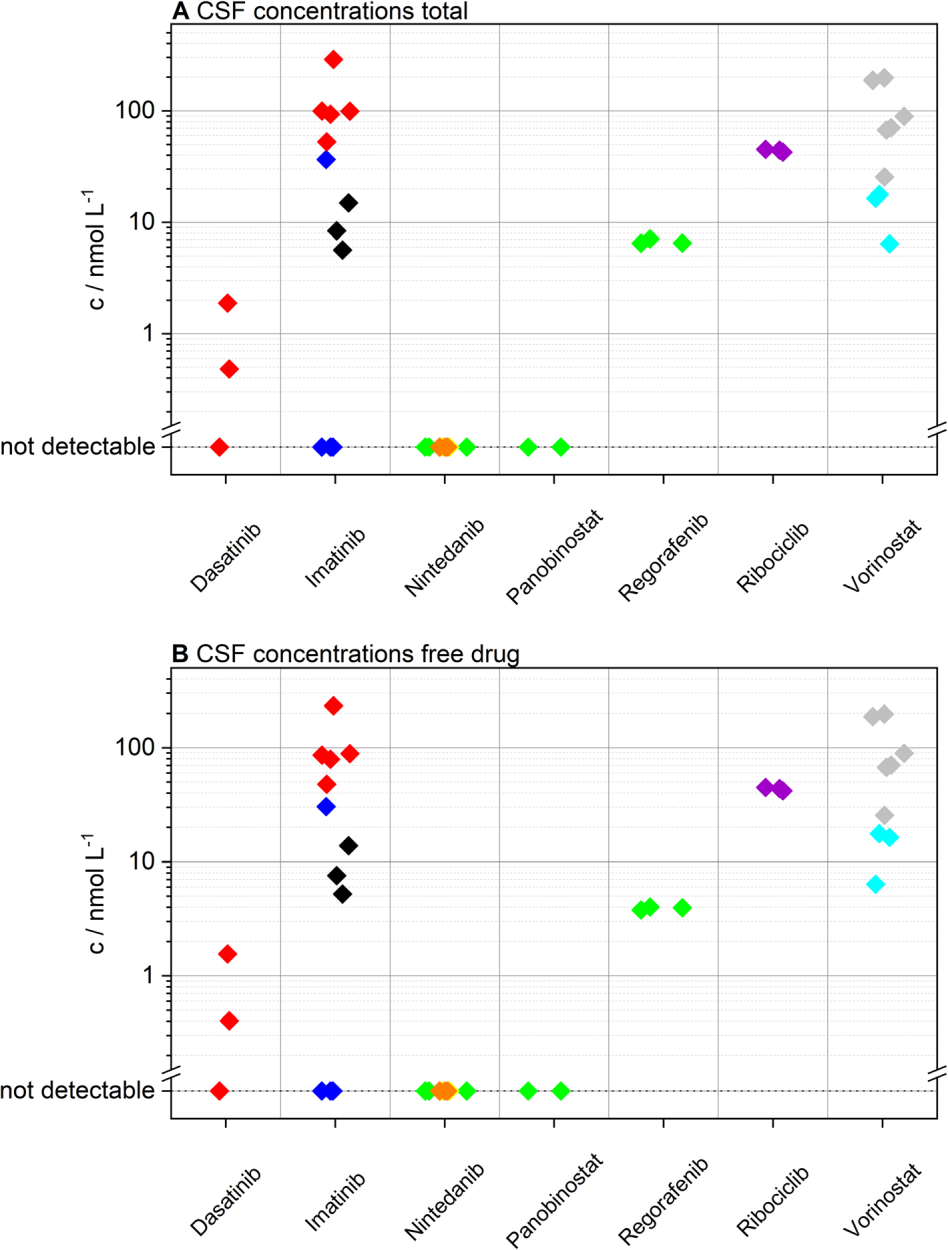
Overview of all analyzed steady-state cerebrospinal fluid (CSF) samples. **a** Total measured und (**b**) calculated free, unbound CSF concentrations of the investigated drugs depicted in nM/L. Individual patients are indicated by the color-coding (Patient #1 red, #2 blue, #3 black, #4 green, #5 yellow, #6 orange, #7 violet, #8 grey, #9 cyan)

In total, 42 CSF samples of 9 pediatric patients (3 female, 6 male) receiving 7 different drugs were analyzed (Fig. 1). Detailed clinical data are outlined in Table 1. Quantitation was successful in 26 CSF specimens, including samples for dasatinib (mean 1.2 nM, range 0.5–1.9 nM), imatinib (mean 77.7 nM, range 5.7–288.5 nM), regorafenib (mean 6.7 nM, range 6.5–7.1 nM), ribociclib (mean 44.1 nM, range 42.6–45.2 nM) and vorinostat (mean 75.4 nM, range 6.4–197.2 nM). Imatinib and dasatinib were detected in 75% (9/12) and 66% (2/3) respectively. In contrast, nintedanib was not identified in any patient sample. To prove oral drug uptake of nintedanib we tested an available matched serum sample for nintedanib at one time point (patient #4), which clearly proved gastrointestinal uptake (30 nM in serum). Normalized concentrations (according to the dosage) were similar to previously published data [47]. Similarly, panobinostat was untraceable in CSF samples in agreement with a previous study [48]. Intrapatient CSF concentrations of each substance were within the same order of magnitude (Fig. 2a). Moreover, samples from different individuals (imatinib, nintedanib, vorinostat) showed no significant interpatient variability (Fig. 2a, Additional file 1: Figures S4 and S5). Interestingly, imatinib was only detected in one out of four samples in one case (#3) (Additional file 1: Figure S4). This sample was obtained at a much later time point than the negative ones. Moreover, we detected much higher CSF protein levels at this time point, suggesting a change of BBB integrity during the course of disease. Consequently, we tested whether CSF protein levels routinely measured at the same time point as drug penetration analyzes correlated to imatinib concentrations and could indeed detect a positive correlation (Additional file 1: Figure S6). In order to exclude protein concentration as potential general confounding factor in our study, we checked for a potential bias in our patient cohort. We could not find any association between detection of substances in CSF and protein levels (Additional file 1: Figure S7).

As a next step, free drug levels were calculated using the law of mass action approach stated in the supplementary, showing that protein binding may be neglected for most of the investigated compounds (see Fig. 2b). In case of dasatinib (mean 1 nM, range 0.4–1.6 nM), ribociclib (mean 43.5 nM, range 41.8–44.8 nM) and vorinostat (mean 75.3 nM, range 6.4–197.1 nM), the unbound fraction accounted for about 99% of the total drug amount measured. Furthermore, the level of unbound imatinib (mean 65.8 nM, range 5.2–233.4 nM) was calculated to be nearly 88% of the total amount. Since regorafenib is highly bound to proteins in serum (99.5% [49]), the amount of protein-bound drug in CSF was not negligible, as less than 60% of regorafenib are freely available in CSF (mean 3.9 nM, range 3.8–4 nM).

### Detection of drug metabolites in patient CSF

**Fig. 3:**
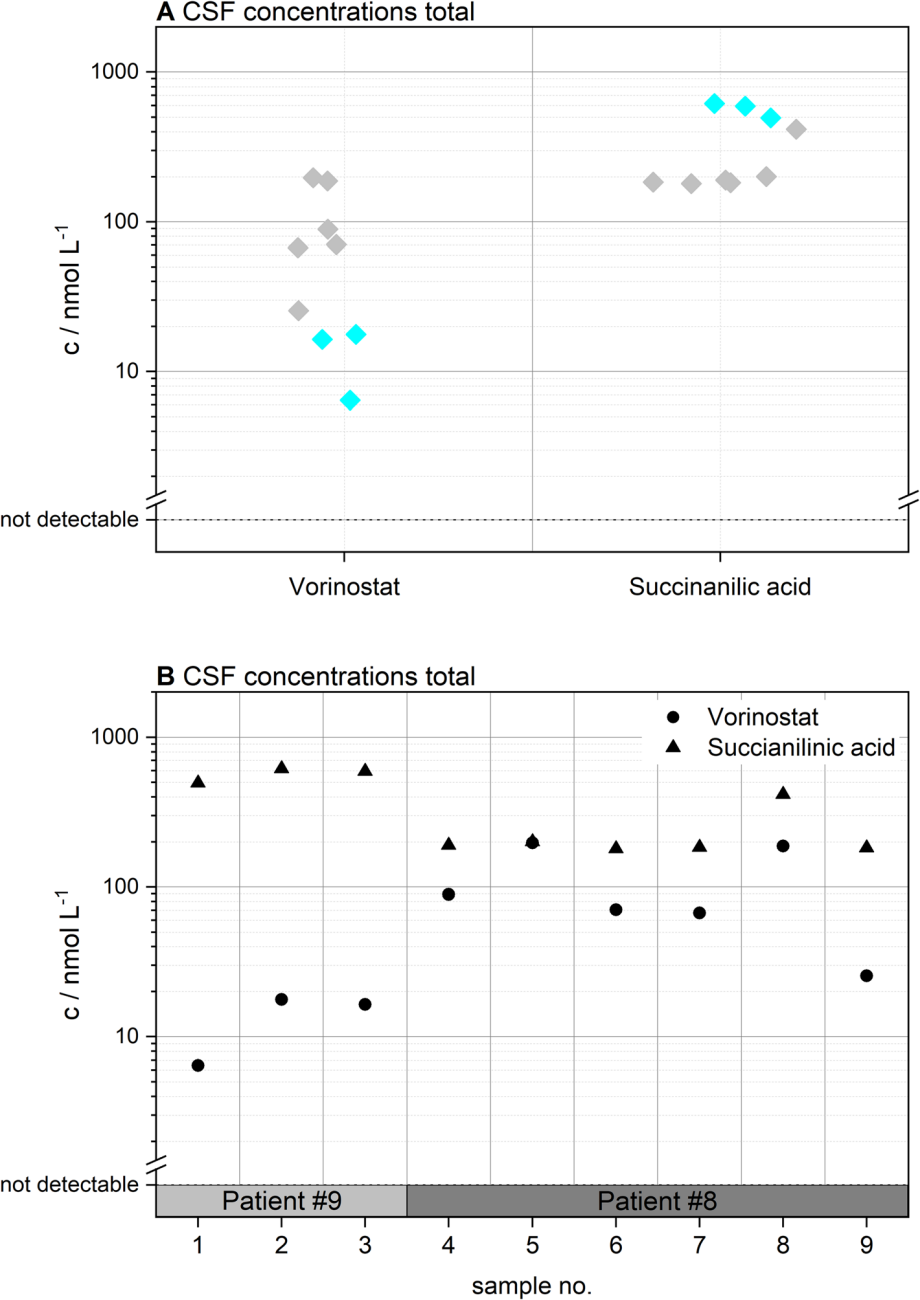
CSF levels of vorinostat and its main metabolite succinanilic acid stratified for (**a**) vorinostat vs. succinanilic acid and (**b**) individual samples. Patient numbers are indicated and given by the color-coding (Patient #8 grey and #9 cyan)

Using high performance liquid chromatography ion mobility quadrupole time-of-flight mass spectrometry (HPLC-IMS-Q-TOF MS) measurements in combination with prediction software and in-house generated databases, it was also possible to evaluate the metabolic fate of the examined substances in a semi-automated way. ADMET Predictor 9.0 and Pallas 3.8 were used to predict metabolites of parent drugs qualitatively, as both software packages provide molecular structure output of P450 metabolized substances. In contrast, Smartcyp 2.4.2 calculates the likelihood of a certain molecular moiety to be metabolized by P450 cytochromes based on characteristics within the molecule’s 2D structure. Detected metabolites and potential biological activity are summarized in Table 2. Metabolites of imatinib (demethylated imatinib (=CGP74588; active), hydroxylated imatinib (=AFN911; inactive), N-glucuronidated imatinib and O-glucuronidated imatinib), ribociclib (demethylated ribociclib (=LEQ803; active) and hydroxylated LEQ803), and vorinostat (succinanilic acid and glucuronidated vorinostat) were identified. In case of vorinostat, it was even possible to quantitate its pharmacologically inactive main metabolite, succinanilic acid [50]. In this context, Fig. 3 shows the concentrations of the parent drug and the metabolite in CSF samples.

**Table 2:** Overview of the detected metabolites in cerebrospinal fluid

Parent Drug	Metabolite	Structure	Active / Inactive
imatinib	demethylated imatinib = CGP74588	known	active
imatinib	hydroxylated imatinib = AFN911	known	unknown
imatinib	N-glucuronidated imatinib	unknown	unknown
imatinib	O-glucuronidated imatinib	known	unknown
ribociclib	demethylated ribociclib = LEQ803	known	active
ribociclib	hydroxylated LEQ803	unknown	unknown
vorinostat	succinanilic acid	known	inactive
vorinostat	glucuronidated vorinostat	known	inactive

### Comparison to established predictors of blood-brain barrier penetration

**Fig. 4:**
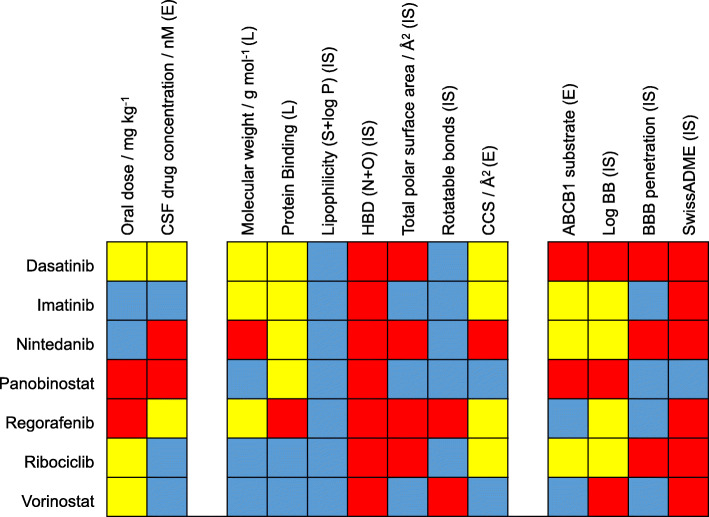
Comparison of real-world data to predictors of blood-brain barrier penetration. Normalized color-coded (red, unfavorable; yellow, intermediate; blue, favorable for BBB penetration) results, summarizing all examined parameters including literature data (L), in silico results (IS) and the quantification results of active ingredients in CSF samples and ABCB1 experiments (E). Detailed color coding: oral dose (red, < 1 mg/kg; yellow, 1–5 mg/kg; blue > 5 mg/kg); CSF concentration (red, not detected; yellow, < 10 nM; blue, > 10 nM); Molecular weight (red, > 500 g/mol; yellow, 450-500 g/mol; blue, < 450 g/mol; Protein binding (red, > 99%; yellow, 90–99%; blue, < 90%); S + logP (red, < 1,5; blue, > 1,5); Hydrogen bond donors (red, > 5; blue < 5); total polar surface area (red, >90A/Å2; blue<90A/Å2). Rotatable bonds (red, > 8; blue, < 8); collision cross section (red, > 250 Å; yellow, 200-250 Å; blue, < 200 Å); ABCB1-substrate (red, > 2 fold-control; yellow, 1–2 fold-control; blue < 1 fold-control); LogBB (= predicted logarithm of the brain/blood concentrations using ADMET predictor) (red < 0; blue > 0); BBB permeation (predicted likelihood of BBB permeation using ADMET predictor, red, low; blue, high) and SwissADME (red, no penetration, blue, penetration). A detailed description of color-coding is provided also provided in supplementary Additional file 1: Table S5.

As preclinical prediction of BBB penetration remains essential for development of improved anti-cancer therapies for brain tumor patients, we sought to validate commonly applied methods for BBB penetrance prediction with our dataset. Figure 4 provides a graphical overview of this comparison. Nintedanib as the largest molecule (MW 539.6 g mol^− 1^) was not detected in CSF, in line with the finding that larger molecules are less frequently found to cross the BBB [17]. Conversely, panobinostat – one of the smallest molecules in our panel – was also not detected. As already mentioned, free drug levels are the essential parameter in order to assess target engagement [4]. Within our drug panel, we could show that high protein binding was the essential property lowering free drug levels in case of regorafenib, which we show to cross the BBB, but showed lower free drug levels. With respect to chemical properties, all molecules showed a favorable profile according to lipophilicity (S + logP), another common predictor for BBB-penetration [17, 19]. Consequently, differences in lipophilicity could not explain the observed differences. In contrast, the amount of hydrogen bond donors showed unfavorable profiles for all drugs also making it unsuitable for BBB penetrance prediction in our panel. The utility of total polar surface area (TPSA) appeared to be mixed as smaller molecules indeed did cross the BBB, however, with the exception of panobinostat. The number of rotatable bonds showed an unfavorable profile for regorafenib and vorinostat which where both found to cross the BBB. We additionally evaluated ABCB1-inhibition as well as intracellular drug accumulation upon ABCB1-inhibition (Additional file 1: Figure S8). Interestingly, ABCB1-inhibition did not necessarily correlate with ABCB1-substrate testing. Consequently, we only used the fold-change of drug accumulation upon ABCB1-inhibition as parameter for ABCB1-affinity. ABCB1 substrate evaluation was most favorable for regorafenib and vorinostat and intermediate for ribociclib, imatinib, and nintedanib. Dasatinib and panobinostat, in contrast, were highly transported by ABCB1 potentially explaining the observed poor penetration of panobinostat. Furthermore, the CCS as novel descriptor of molecular branching and volume was included based on HPLC-IMS-Q-TOF MS analyses. The finding that imatinib and regorafenib show a significantly lower CCS value than would be expected from the MW, and thus are much more compact molecules (see Additional file 1: Figure S3), supported their permeation of the blood-CSF barrier and their pronounced presence in the CSF.

Last, we aimed at the evaluation of commonly applied in silico methods for the prediction of BBB penetration. These algorithms integrate multiple of the aforementioned parameters and are therefore generally considered as more advanced and reliable tools for evaluation of BBB penetrance [44, 51]. A comparison to our patient-derived dataset, however, showed mixed results. ADMET Predictor correctly predicted BBB penetration properties for imatinib, nintedanib and regorafenib and LogBB of vorinostat, but failed for the other drugs. The second evaluated algorithm, SwissADME, only predicted panobinostat to penetrate the BBB. However, we did not detect panobinostat, but other compounds in the CSF.

## Discussion

The integration of molecular tumor profiling to detect oncogenic dependencies and the availability of multiple targeted compounds for the respective alterations has widely improved anti-cancer treatment. However, low CNS penetration of small molecule inhibitors caused by the BBB poses a major obstacle for the clinical use of improved and innovative therapies against brain tumors. Although various in vitro, in silico and in vivo models have been developed to predict BBB drug penetration in the past decades, the true nature of the BBB in CNS tumors and the actual penetrance of drugs into the malignant tissue is still difficult to assess. Consequently, it frequently remains difficult to clarify whether the failure of promising in vitro approaches in clinical trials is owed to a poor anti-tumor effect or limited CNS penetration. As the blood-CSF barrier is composed of similar structures as the BBB and CSF is directly connected to the CNS tissue, measuring CSF levels is widely considered as feasible way to indicate BBB penetration [52]. However, CSF sampling in the majority of studies is mostly restricted to inter-operative collection or lumbar puncture, both procedures with certain risks and inaccuracies. We have already shown that treatment and CSF collection via Ommaya-reservoirs is a safe procedure in pediatric patients with brain tumors [53]. In this study, we developed a reliable method to detect and quantify traceable amounts of oral anti-cancer compounds in pediatric patients receiving intrathecal therapy. Being aware of the limitation that almost no corresponding serum samples and no specific time points were available for our retrospective study, we first evaluated CSF levels across different time points at steady state and did not detect any significant differences. By these means, we show for the first time that CSF sampling from Ommaya reservoirs represents a feasible, reproducible and reliable method for CNS penetrance ascertainment. It has to be noted, that most of the studied cases exhibited leptomeningeal disease, which also might influence BBB penetration resulting in a less effective blood-CSF barrier. This is also suggested by our finding that CSF penetrance might change during the course of disease. In the single case without leptomeningeal disease within our cohort, nintedanib could not be detected in the CSF (case #5). Nintedanib was, however, also not measurable in the two other cases (#4 and #6), suggesting that presence of leptomeningeal disease might be of lower significance as compared to other parameters, at least within our small study cohort. Nevertheless, drug concentrations in CSF may be of importance in particular for the treatment of leptomeningeal disease. Moreover, the study collective comprises different tumor entities which also might bias the comparison of the investigated compounds. Still, we did not observe differences with respect to tumor entities within our limited dataset. It is also worth noting that all patients received concomitant systemic and intrathecal therapy potentially influencing the permeability of the blood-CSF barrier. As indicated in Additional file 1: Table S1 all but one case were treated according to a modified MEMMAT protocol [36]. Therefore, our cohort did not exhibit profound differences with respect to concomitant systemic treatment. With respect to intrathecal therapy, the observed interpatient differences could also not be explained by different concomitant medication. For imatinib, all patients received only VP-16 and in case of vorinostat one patient received VP-16, cytarabine, and topotecan, whereas the other case was treated with VP-16, liposomal cytarabine, and methotrexate (Additional file 1: Table S1). However, vorinostat levels were slightly lower in the latter case (Fig. 2, case #9 cyan) although one would rather expect liposomal cytarabine or methotrexate to alter permeability of the blood-CSF barrier. We further included testing of cases who sequentially received more than one of the investigated compounds (Additional file 1: Table S2, patients #1 and #4). Interestingly, of the three drugs investigated in case #4, only regorafenib could be detected, whereas nintedanib and panobinostat were not measurable. Notably, regorafenib was the second drug within this sequential treatment, again corroborating our notion that prior treatment and interindividual differences were only a minor confounder in our study.

In detail, we could detect significant levels of dasatinib, imatinib, regorafenib, ribociclib, and vorinostat in CSF of brain tumor patients. In contrast, nintedanib and panobinostat were not detected albeit only one case was available for panobinostat sampling. With respect to panobinostat, it is also worth noting that due to the age of the patient (19 years, patient #4) the treatment dosing was chosen according to the regimen approved for hematological malignancies in adults (20 mg, 3 doses / week). Consequently, the chosen dose was lower than in recent studies within the pediatric population [54]. Corroborating our results panobinostat levels were also below the detection limit in a recently published study in children using higher panobinostat doses [54]. Comparing our quantitation results to serum-levels from former studies, CSF-levels of dasatinib [55], imatinib [56], regorafenib [57], ribociclib [58] and vorinostat [59] showed significant differences (multiple orders of magnitude). The approach of evaluating CNS penetration via CSF sampling has been questioned [60] and studies directly measuring drug penetrance into tumor tissue are generally favored. In this context it is worth noting that overall comparison of our data to preclinical animal studies for dasatinib [61] and regorafenib [62] as well as a clinical trials with ribociclib [63–65] in glioblastoma patients demonstrated comparable results.

The CSF-levels of dasatinib were in the low nM range which is well in agreement with CSF levels described for this tyrosine kinase inhibitor in acute lymphoblastic leukemia (ALL) patients [66]. These results are also in agreement with preclinical studies on brain and tumor penetration of dasatinib in mice [61, 62], which also described the influence of ABCB1 and ABCG2. With respect to biological activity, the detected dasatinib levels were approximately 10-fold below the IC_50_ determined for high-grade glioma [67]. The sample in our study, in contrast, was derived from a patient being treated for a recurrent CNS germ cell tumor, which has been suggested as promising therapeutic approach [68] but preclinical in-vitro data determining the IC_50_ for germ cell tumors are still lacking.

Similarly, levels of imatinib were in the same magnitude as described for an ALL patient [69]. In contrast to dasatinib, the detected imatinib levels were in the range of the IC_50_ for certain high-grade glioma models [67]. Interestingly, one case in our cohort was treated with imatinib for a high-grade glioma (#2), but in vitro data is lacking for the other two entities plexus carcinoma and CNS germ cell tumor.

Regorafenib, another kinase inhibitor with promising clinical activity against brain tumors [70], also reached detectable levels in CSF of one patient. Moreover, regorafenib CSF levels were in the same magnitude as detected in brain tissue within preclinical animal studies [60]. However, free drug levels were markedly reduced due to its high protein binding properties and were not in the range of the IC_50_ previously determined for other pediatric tumor models [71].

Ribociclib, a CDK4/6 inhibitor was also detected at clinically relevant levels in CSF. The magnitude was comparable, albeit lower compared to recently published studies [64, 65] investigating ribociclib levels in CSF and tumor tissue of adult glioblastoma patients. The respective studies reported levels from 374 to 630 nM compared to 43 nM detected in our patient. It is worth noting, that the investigated dose in these studies was 900 mg per day in adults (approximately 11 mg/kg), which is distinctly higher than in our pediatric patient treated with 3.33 mg/kg daily. Furthermore, our observation was in line with results from preclinical mouse studies [63]. In general, ribociclib CNS levels were in the therapeutic range determined in vitro for neuroblastoma cell lines [64] but data on brain tumors or epithelioid sarcomas as in our cohort are still lacking.

We further show that the histone deacetylase inhibitor vorinostat penetrates the CSF. The concentration, however, was about 10-fold lower as compared to IC_50_ values in preclinical models of medulloblastoma [72].

Using in silico based metabolite prediction approaches and high-resolution mass spectrometry, the metabolic fate of the target analytes in CSF was monitored for the first time. Although predominantly phase I metabolites such as oxidation, hydroxylation and hydrolysis products were detected, their existence in CSF is an exciting finding, since especially phase one metabolites, which we detected for imatinib and ribociclib, might still be pharmacologically active [73, 74]. Additionally, the main metabolite of vorinostat, succinanilic acid, was found in CSF samples at high concentrations. In conclusion, we show that the combination of in silico based metabolite prediction approaches and high-resolution mass spectrometry is a highly versatile tool in drug metabolite analysis, which may also have a therapeutic effect.

Last, we sought to evaluate the obtained patient data with respect to physicochemical properties of the respective small molecules and commonly applied predictors of BBB penetration comprehensively. We confirm that low MW, low protein binding and low ABCB1-affinity as in case of vorinostat and ribociclib are favorable predictors of BBB-penetration. It has to be noted that unfavorable characteristics for only one of the parameters may already lead to significantly lower CSF concentrations as in case of panobinostat, which exhibits low MW and moderate protein binding but high ABCB1-affinity. Moreover, we evaluated the in silico tools SwissADME and ADMET Predictor which integrate multiple physicochemical properties to predict BBB penetration of molecules. Interestingly, there was no or only a poor correlation to the findings in our data. This highlights the overall complexity of prediction BBB penetration as multiple physicochemical and biological factors determine the permeability of the BBB barrier. It might also reflect that ABCB1-affinity is difficult to predict in silico and that preclinical evaluation should be based on a combination of physicochemical and biological tests. Taken together, our comparative analyzes emphasize the necessity to evaluate the BBB barrier penetration in clinical studies.

## Conclusions

In summary, we present CSF-sampling via Ommaya-reservoirs as a feasible, reliable and safe strategy to investigate CSF-concentrations of small molecule inhibitors in brain tumor patients. This method could open new opportunities to evaluate BBB penetration in patients with brain tumors in future clinical trials. Finally, our correlations of patient-derived data to established predictors of CNS-penetration emphasize the importance of a comprehensive approach for preclinical assessments, taking into account both biological and physicochemical properties.

## Supplementary information

**Additional file 1:Table S1.** Supplementary information on clinical parameters and sample collection. **Table S2.** Sequential therapies of the investigated drugs in the study cohort. **Figure S1.** Depiction of the used HPLC gradient for sample analysis. **Table S3.** Overview of the used mass spectrometer parameters. **Figure S2.** Normalized chromatogram of a standard solution of dasatinib, imatinib, nintedanib, panobinostat, regorafenib, ribociclib, and vorinostat obtained with the developed HPLC-QqQ MS method. **Table S4.** Overview of the corresponding method performance parameters. **Figure S3.** Depiction of measured CCS values of dasatinib, imatinib, nintedanib, panobinostat, regorafenib, ribociclib, and vorinostat. **Figure S4.** Depiction of the quantitation results for imatinib CSF samples sorted by patient. **Figure S5.** Depiction of the quantitation results for vorinostat CSF samples sorted by patient. **Figure S6.** Depiction of the correlation between imatinib levels and CSF protein amounts. **Figure S7.** Matched CSF protein concentrations of all investigated CSF samples stratified for drug and individual patients. **Figure S8.** ABCB1 testing. **Table S5.** Supplementary description of the color-coding used in the Fig. 4 of the manuscript.

## Data Availability

All data generated or analyzed during this study are included in this published article.

## Abbreviations

BBB: Blood-brain barrier
CCS: Collision cross section
CNS: Central nervous system
CSF: Cerebrospinal fluid
HPLC-MS: High performance liquid chromatography mass spectrometry
HPLC-IMS-Q-TOF MS: High performance liquid chromatography ion mobility quadrupole time-of-flight mass spectrometry
HBA: Hydrogen-bond acceptor
HBD: Hydrogen-bond donor
FACS: Fluorescence-activated cell sorting
LOD: Limit of detection
MRI: Magnetic resonance imaging
MW: Molecular weight
PSA: Polar surface area
RSD: Relative standard deviation
TPSA: Total polar surface area
