# Supplementary material for: Cerebrospinal fluid penetration of targeted therapeutics in pediatric brain tumor patients
Source: Acta Neuropathol Commun. 2020 Jun 3;8:78. doi: 10.1186/s40478-020-00953-2 (PMC7268320; doi:10.1186/s40478-020-00953-2)
Supplement: Supplementary file 1 — Additional file 1:Table S1. Supplementary information on clinical parameters and sample collection. Table S2. Sequential therapies of the investigated drugs in the study cohort. Figure S1. Depiction of the used HPLC gradient for sample analysis. Table S3. Overview of the used mass spectrometer parameters. Figure S2. Normalized chromatogram of a standard solution of dasatinib, imatinib, nintedanib, panobinostat, regorafenib, ribociclib, and vorinostat obtained with the developed HPLC-QqQ MS method. Table S4. Overview of the corresponding method performance parameters. Figure S3. Depiction of measured CCS values of dasatinib, imatinib, nintedanib, panobinostat, regorafenib, ribociclib, and vorinostat. Figure S4. Depiction of the quantitation results for imatinib CSF samples sorted by patient. Figure S5. Depiction of the quantitation results for vorinostat CSF samples sorted by patient. Figure S6. Depiction of the correlation between imatinib levels and CSF protein amounts. Figure S7. Matched CSF protein concentrations of all investigated CSF samples stratified for drug and individual patients. Figure S8. ABCB1 testing. Table S5. Supplementary description of the color-coding used in the Fig. 4 of the manuscript. [file 40478_2020_953_MOESM1_ESM.pdf]

Cerebrospinal fluid penetration of targeted therapeutics in pediatric brain tumor patients

Supplementary Material

Armin Sebastian Guntner[1], Andreas Peyrl[2,3], Lisa Mayr[2,3], Walter Berger[3,4], Irene Slavc[2,3], Wolfgang Buchberger[1], Johannes Gojo[2,3,4]

1 Institute of Analytical Chemistry, Johannes Kepler University, Linz, Austria

2 Comprehensive Cancer Center-Central Nervous System Tumors Unit, Medical University of Vienna, Austria

3 Department of Pediatrics and Adolescent Medicine, Medical University of Vienna, Austria

4 Institute of Cancer Research, Department of Medicine I, Medical University of Vienna, Austria

Corresponding author:

Johannes Gojo
johannes.gojo@meduniwien.ac.at
Institute of Cancer Research, Department of Medicine I, Medical University of Vienna, Austria
Währinger Gürtel 18-20
1090 Vienna

Funding:

The work was supported by the „Verein unser_kind "and the Austrian Science Fund FWF (project number: P30105 to W.B.).

Conflict of interest:

The authors have no conflict of interest to declare.

Table of Content:

1. Detailed Outiline on Clinical Parameters and Sample Collection 3
2. Development of an HPLC-MS method for the bioanalysis of dasatinib, imatinib, nintedanib, panobinostat, regorafenib, ribociclib and vorinostat 4
3. Collision cross sections of dasatinib, imatinib, nintedanib, panobinostat, regorafenib, ribociclib and vorinostat 10
4. Calculation of free drug levels... 11
5. Protein levels in patient samples... 12
6. Experiments with ABCB1-overexpressing KBC1 cells 15
7. Color-coding in Figure 4 of the Manuscript 16

1. Detailed Outiline on Clinical Parameters and Sample Collection

In the following Tables (S1 and S2) detailed information on patient therapy and sample collection are given.

Table S1 Supplementary information on clinical parameters and sample collection.

Drug	Patient #	Number of samples	period of CSF sample collection (weeks)	concomitant medication oral/i.v.	concomitant medication i.th. (alternating)
Imatinib	1	5	31	MEMMAT	VP-16
	2	3	1	-	VP-16
	3	4	9 (1 sample 5 years later at re-initiation of treatment)	MEMMAT	VP-16
Dasatinib	1	3	5	MEMMAT	VP-16
Nintedanib	4	4 (+1 matched serum at one time point)	6 (1 sample 1 year later at re-initiation of treatment)	MEMMAT	VP-16 topotecan liposomal cytarabine
	5	3	2	MEMMAT (without bevacizumab)	VP-16 cytarabine
	6	3	6	MEMMAT (incl. alternating oral temozolomide 50mg/m²)	VP-16 topotecan liposomal cytarabine
Panobinostat	4	2	3	MEMMAT (without oral VP-16/cyclophosphamide)	VP-16 liposomal cytarabine
Regorafenib	4	3	2	MEMMAT	VP-16 liposomal cytarabine
Ribociclib	7	3	4	MEMMAT	VP-16 topotecan
Vorinostat	8	6	4	MEMMAT	VP-16 topotecan cytarabine
	9	3	1	MEMMAT	VP-16 liposomal cytarabine methotrexate

Table S2 Sequential therapies of the investigated drugs in the study cohort. The period to the prior drug is indicated in brackets.

Patient #	1st	2nd	3rd	4th
1	imatinib	dasatinib (10 weeks)	-	
2	imatinib	-	-	
3	imatinib	-	-	
4	nintedanib	regorafenib (31 weeks)	panobinostat (10 weeks)	nintedanib (5 weeks, re-initiation)
5	nintedanib	-		
6	nintedanib	-		
7	ribocilib	-		
8	vorinostat	-		
9	vorinostat	-		

2. Development of an HPLC-MS method for the bioanalysis of dasatinib, imatinib, nintedanib, panobinostat, regorafenib, ribociclib and vorinostat

Dasatinib, imatinib, nintedanib, panobinostat, regorafenib, ribociclib, vorinostat were purchased from Selleckchem (Houston, TX, USA). Standards were originally prepared as a 10 mM DMSO solution, but were further diluted with methanol to obtain stock solutions containing analyte concentrations of 10 mg L^{-1} and were stored at 4 °C. The internal standard (ISTD) carbamazepine (> 98 %) was obtained from Sigma-Aldrich Handels GmbH (Vienna, Austria) and used as a methanolic stock solution with a concentration of 2 g L^{-1}, which was diluted as needed.

Succinanilic acid (p.a.) was purchased from Sigma-Aldrich Handels GmbH (Vienna, Austria) diluted to a 400 mg L^{-1} methanolic stock solution and stored at 4 °C.

All used solvents were HPLC grade and were purchased from VWR International GmbH (Darmstadt, Germany).

Formic acid (≥ 96 %) was purchased from Sigma-Aldrich Handels GmbH (Vienna, Austria).

Water was used in Millipore quality obtained from a Millipore purification system (Molsheim, France).

CSF samples (< 0.5 mL) of pediatric patients receiving cancer therapy drugs were collected using an Ommaya reservoir, and were stored at -80 °C prior to further analysis.

Sample preparation included protein precipitation (100 µL serum or CSF + 300 µL methanol containing the internal standard carbamazepine). Although protein levels in CSF are significantly lower compared to serum, precipitation is needed in order to avoid precipitation of proteins within the HPLC system resulting in shortened lifetime of analytical columns and other instrument parts. Proper homogenization of sample material was achieved using a Thermoshaker Thermal Shake lite (1500 rpm, 10 min, VWR International GmbH, Darmstadt, Germany). Subsequently the samples were centrifuged using a

Megafuge 1.0 (4000 rcf, 10 min, Heraeus Instruments, Hanau, Germany) and the supernatant was collected in 200 µL HPLC vial inserts.

The separation of analytes and CSF matrix compounds was achieved by the means of high performance liquid chromatography (HPLC) using a 1200 Series HPLC purchased from Agilent Technologies (Santa Clara, California) combined with a C18 Nucleoshell column (150 x 3 mm; 2.7 µm; endcapped silica) from Macherey-Nagel (Düren, Germany) coupled to a 6420 triple quadrupole mass spectrometer (QqQ MS) or a 6560 Ion-Mobility Quadrupole Time-of-Flight MS (IMS-Q-TOF MS) both from Agilent Technologies (Santa Clara, California).

High performance liquid chromatographic separation was accomplished using an octadecyl reversed phase column in combination with a gradient mobile phase of methanol and 1 vol.% formic acid. In this context, Figure S1 gives detailed information on the developed chromatographic gradient system.

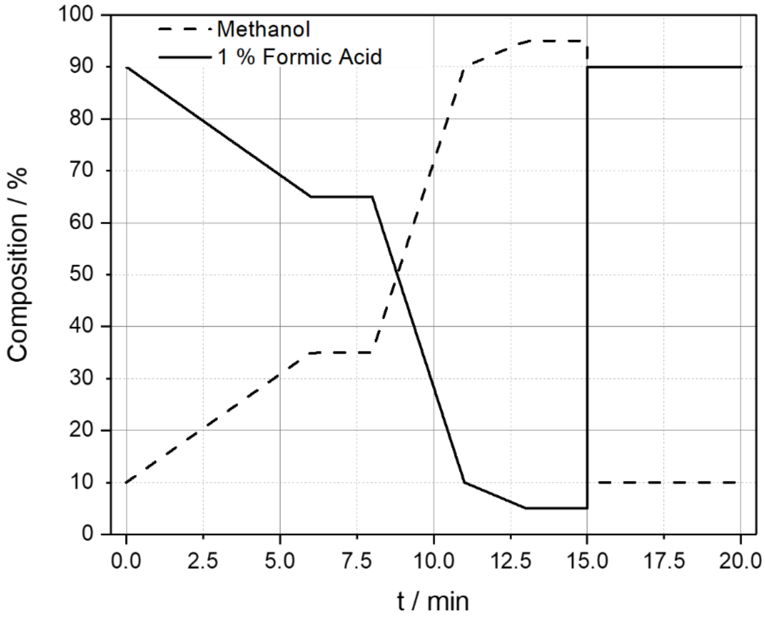

Figure S1 Depiction of the used gradient system within the multi-method to separate all mentioned analytes as well as matrix components.

In all corresponding analyses, a flow rate of 0.6 mL min^{-1} at a column compartment temperature of 50 °C and an injection volume of 5 µL was used.

HPLC-QqQ MS hyphenation by the means of positive electrospray ionization was used for quantitational purposes. Hereby, a gas flow of 11 L min^{-1} at a temperature of 300 °C, a nebulizer pressure of 15 psi and a capillary voltage of 4 kV were used within the source of the Triple Quadrupole instrument for proper ionization of target analytes. Additionally, as part of method development, selected reaction monitoring transitions were investigated for each analyte to ensure sensitivity and selectivity. The most abundant transitions were used as quantifier and at least one lower abundant product ion was used as qualifier. Quantifier/qualifier ion ratios were then used to ensure selectivity.

Data processing was achieved using MassHunter Workstation Software B.05.02 from Agilent Technologies. In this context, Table S3 shows the used dynamic multiple reaction monitoring method for quantitation, including all transitions and corresponding collision energies as well as fragmentor voltages and retention times.

Table S3 Overview of the used QqQ transitions for quantitative and qualitative characterization in Dynamic MRM Mode using 7 V of cell acceleration voltage, 500 ms cycle time and positive polarization within an Agilent 6420 QqQ.

Compound Group	Precursor Ion / m/z	Product Ion / m/z	r.t. / min	Fragmentor / V	Collision Energy / eV
Carbamazepine (= internal standard)	237.1	194.2	12.3	102	18
	237.1	165.1	12.3	102	50
	237.1	121.1	12.3	102	26
Dasatinib	488.17	232.1	10.6	168	46
	488.17	193.1	10.6	168	70
	488.17	161	10.6	168	66
Imatinib	494.27	394.2	7.9	164	26
	494.27	247.1	7.9	164	54
	494.27	217.2	7.9	164	22
Nintedanib	540.26	113.1	12	180	26
	540.26	70.2	12	180	58
	540.26	42.2	12	180	60
Panobinostat	350.2	158.2	7.4	102	14
	350.2	143.1	7.4	102	54
Regorafenib	483.09	288.1	13.7	170	26
	483.09	270.1	13.7	170	38
	483.09	202	13.7	170	46
Ribociclib	435.3	322.1	6.6	174	38
	435.3	294.1	6.6	174	46
	435.3	252.1	6.6	174	58
Vorinostat	265.16	232.1	9.7	93	10
	265.16	77.1	9.7	93	58
	265.16	55.1	9.7	93	42

Metabolite detection and determination of collision cross sections was performed using an IMS-Q-TOF MS within a mass range of 100 – 1700 m/z in QTOF-only and IMS mode respectively. Ionization was again obtained by the means of positive electrospray ionization using 5 L min^{-1} of nitrogen stream with 300 °C for drying, a capillary voltage of 3.5 kV and a nebulizer pressure of 35 psig. Further parameters included a sheath gas flow of 11 L min^{-1} at 350 °C and a nozzle voltage of 1 kV. The fragmentor voltage was set to 400 V. Ion mobility experiments were performed with a maximal drift time of 60 ms at a frame rate of 0.9 frames per second resulting in a total of 600 TOF-transients within one frame. Data processing was achieved using IM-MS Browser B.08.00 from Agilent Technologies on the basis of ion

mobility feature extraction and database-assisted Qualitative Workflows B.08.00 Software from Agilent Technologies.

The method validation of the HPLC-QqQ MS method included recovery analysis, linearity testing and determination of lower limits of detection. For that purpose, multiple calibrators were used to test the linearity of the method (including sample preparation) in a range of 800 ng L^{-1} up to 250 µg L^{-1}. The lower limit of detection was defined as the lowest concentration giving a signal with a signal-to-noise ratio of 3. The lower limit of quantification was defined at a signal-to-noise ratio of 10. Intra-day precision was tested on the basis of 5 consecutive measurements of model samples and inter-day precision was determined as the relative standard deviation on average between 2 quality control sets on individual days.

Overall process efficiency (taking into account recovery and matrix effects) was tested by the addition of spike solutions of pre-determined concentration to blank serum or CSF (10 µL + 90 µL), followed by protein precipitation and HPLC-QqQ MS analysis and comparison of resulting signal intensities with standard solutions of known concentrations. Matrix-matched calibration was eventually used to overcome matrix induced suppression/enhancement effects during electrospray ionization.

The developed HPLC-QqQ MS multi-method to quantitate dasatinib, imatinib, nintedanib, panobinostat, regorafenib, ribociclib and vorinostat in cerebrospinal fluid samples was proven robust, reproducible and reliable over a broad range of analyte concentration levels (800 ng L^{-1} to 250 µg L^{-1}).

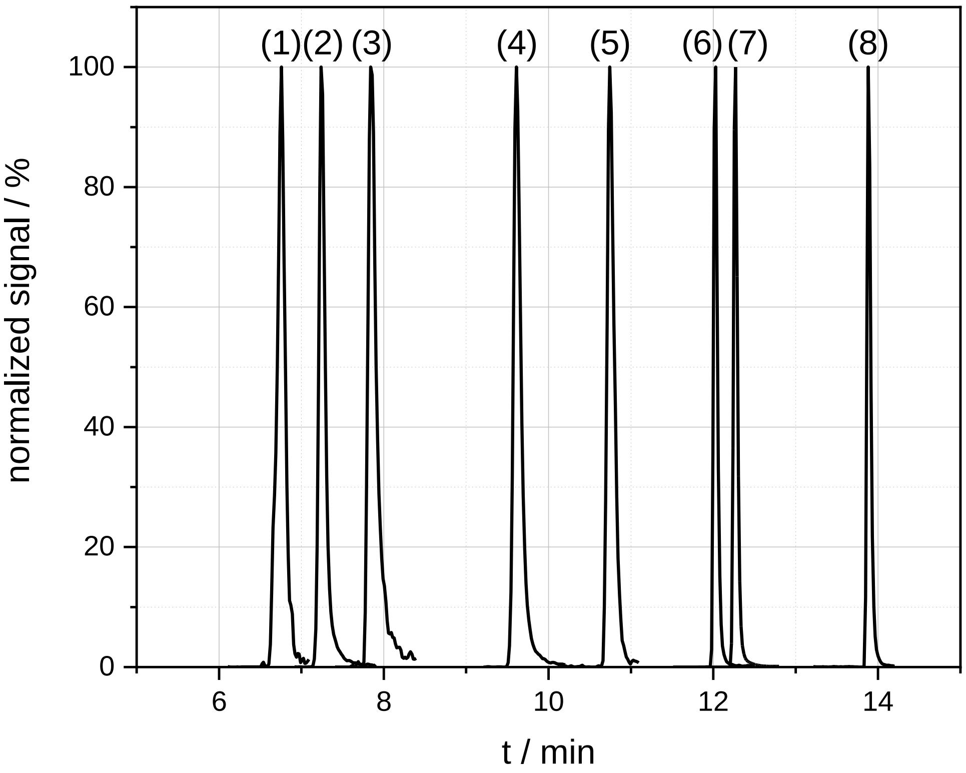

Figure **S2** shows in this context a representative chromatogram of a mixed standard solution.

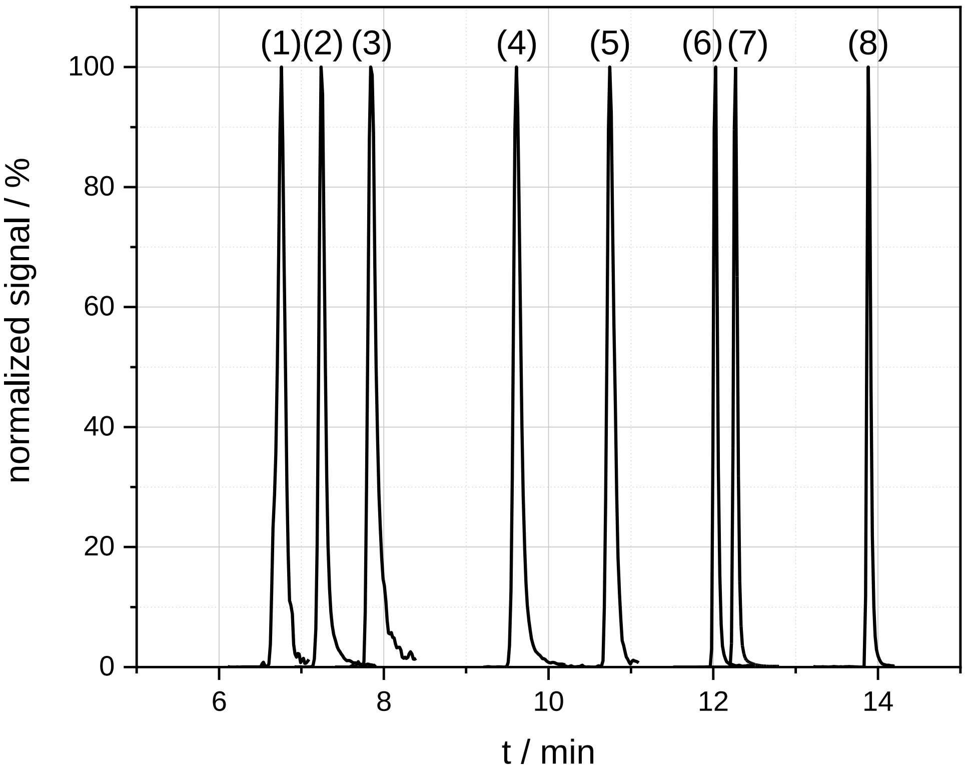

Figure S2 Normalized chromatogram of a 5 µg L^{-1} mixed standard solution obtained with the developed HPLC-QqQ MS multi-method. The displayed peaks are according to the order of elution: ribociclib (1), panobinostat (2), imatinib (3), vorinostat (4), dasatinib (5), nintedanib (6), carbamazepine (7) and regorafenib (8).

Intra-day and inter-day precision were determined to be ≤ 10 % for all analytes. Additionally the developed HPLC-QqQ MS method showed good linearity ($R^2 \geq 0.99$) in a range between the lower limit of detection and 250 µg L^{-1}. The lower limit of detection (LOD) of the method was determined to be ≤ 0.8 µg L^{-1} for all analytes, as shown in detail in Table S4.

.

Table S4 Overview of the precision and lower limits of detection for dasatinib, imatinib, nintedanib, panobinostat, regorafenib, ribociclib and vorinostat in human sample material obtained by the developed HPLC-QqQ MS multi-method.

Substance	Intra-day Precision	Inter-day Precision	LOD / µg L^{-1}
Dasatinib	8.3%	8.5%	0.18
Imatinib	3.1%	8.1%	0.80
Nintedanib	4.4%	8.6%	0.09
Panobinostat	2.8%	3.6%	0.15
Regorafenib	5.5%	7.3%	0.39
Ribociclib	3.7%	4.2%	0.63
Vorinostat	4.0%	4.1%	0.26

In addition to the quantification of the mentioned pharmaceuticals in human sample materials, HPLC-IMS-Q-TOF MS measurements were used in combination with prediction software and in-house generated databases to test for metabolites. In this context, Table 2 of the manuscript gives detailed information of the detected metabolites.

3. Collision cross sections of dasatinib, imatinib, nintedanib, panobinostat, regorafenib, ribociclib and vorinostat

The determination of collision cross sections was performed by the means of HPLC-IMS-Q-TOF MS showing highly reproducible results with intra-day precisions ≤ 0.1 % RSD of 5 consecutive measurements and inter-day precisions ≤ 0.2 % RSD for all analytes. Additionally a positive linear correlation between collision cross sections and mass-to-charge ratio was observed for all analytes excluding imatinib and regorafenib. In this context, Figure S3 gives detailed information on the detected collision cross sections in dependence of the mass-to-charge ratio.

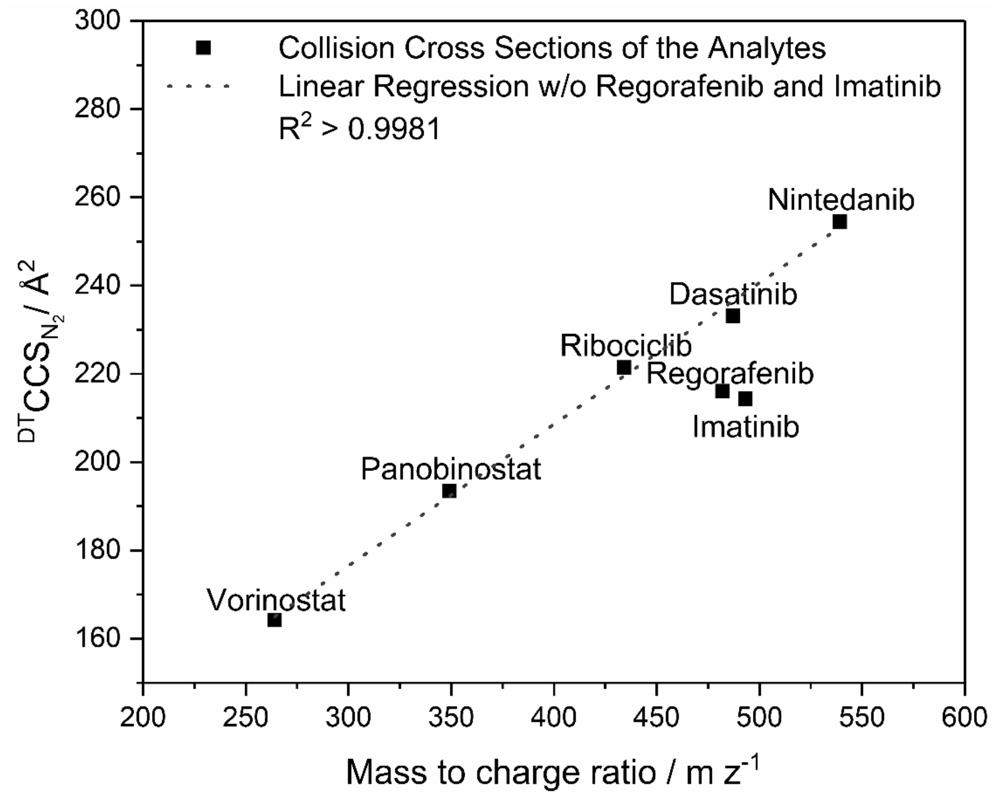

Figure S3 Depiction of measured CCS values of dasatinib, imatinib, nintedanib, panobinostat, regorafenib, ribociclib, and vorinostat.

4. Calculation of free drug levels

The free level of a drug may be calculated, as documented in the work of Avery et al. [38]. Accordingly, following mathematical derivation is feasible:

$$\underset{P - C_B}{Protein} + \underset{C_U}{Drug} \overset{K_D}{\Leftrightarrow} \underset{C_B}{Protein \equiv Drug} \quad (1)$$

P reflects hereby the amount of protein within a matrix. C_U, C_B and C_T describe unbound, bound and total drug concentrations, where:

$$C_T = C_U + C_B \quad (2)$$

and

$$C_U = f_U * C_T \quad (3)$$

including an unbound fraction parameter f_U, which can be calculated. This is possible, because protein concentrations of CSF and serum are available in the literature, but should be determined for individual samples to ensure validity of following predictions. Besides, also a drug's serum protein binding is typically available.

In addition, the therapeutic concentration of drugs is usually significantly lower than the present protein levels within the investigated compartments. Consequently C_B becomes negligible in Equation 4, so K_D can be calculated using serum data.

$$K_D = \frac{(P - C_B) * C_U}{C_B} = \frac{P * C_U}{C_T - C_U} = \frac{P}{C_T / C_U - 1} \quad (4)$$

If K_D is known, the unbound fraction parameter f_U becomes predictable for CSF according to:

$$f_U = \frac{(C_T - K_D - P) + \sqrt{(C_T - K_D - P)^2 + 4K_D C_T}}{2C_T} \quad (5)$$

5. Protein levels in patient samples

In the following vorinostat and imatinib levels are shown as a function of the individual (see Figure S4 and S5).

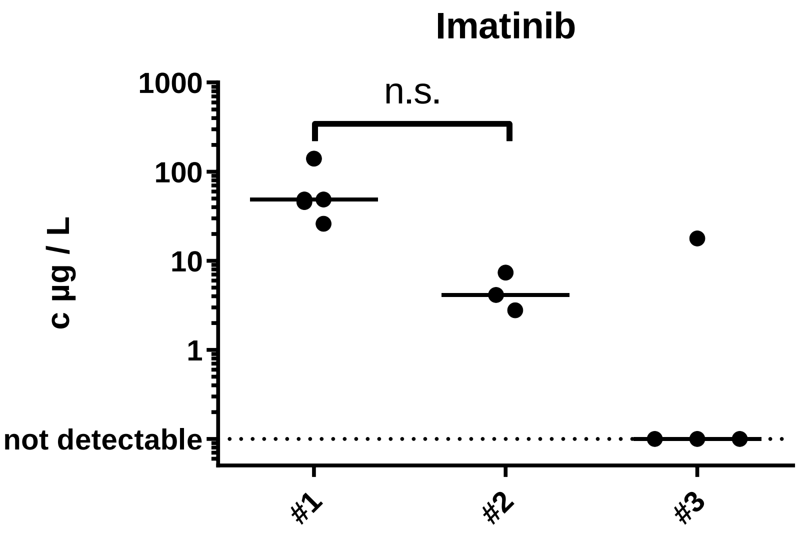

Figure S4 Depiction of the quantitation results for imatinib CSF samples sorted by patient.

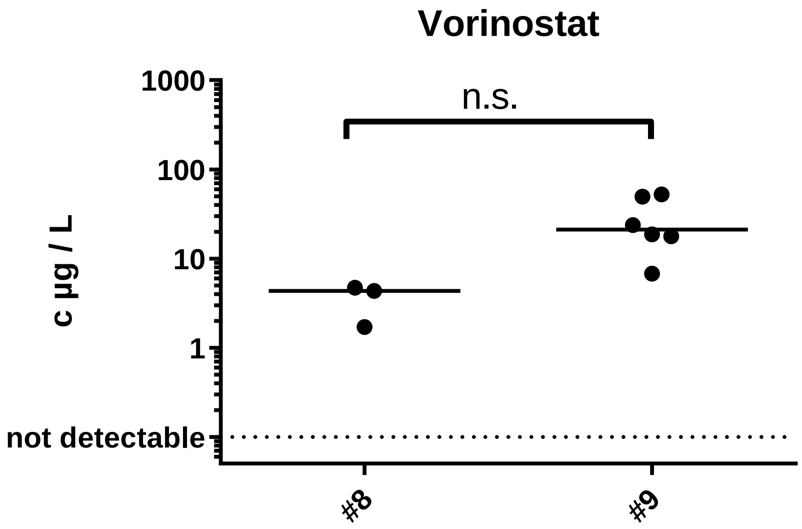

Figure S5 Depiction of the quantitation results for vorinostat CSF samples sorted by patient.

In addition, a possible correlation between imatinib levels and the amounts of CSF proteins was tested, as shown in further detail in Figure S6-S7.

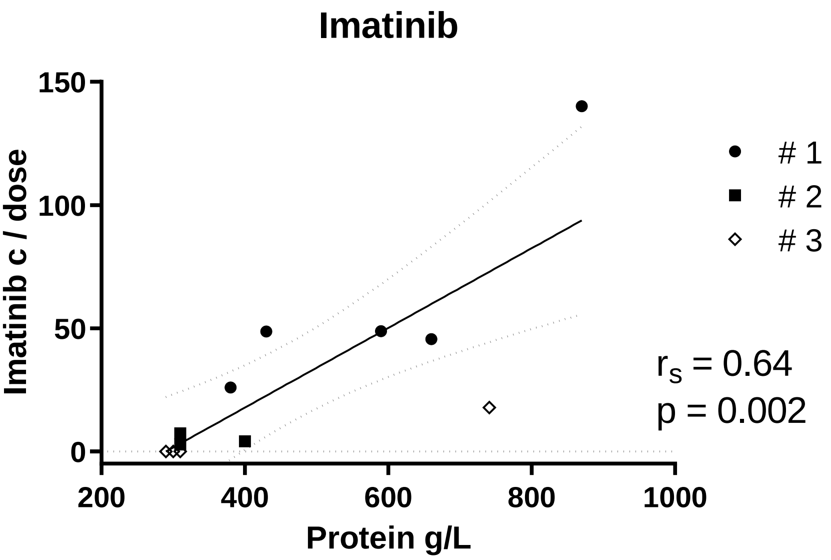

Figure S6 Depiction of the correlation between imatinib levels (µg/L) and CSF protein amounts (g/L).

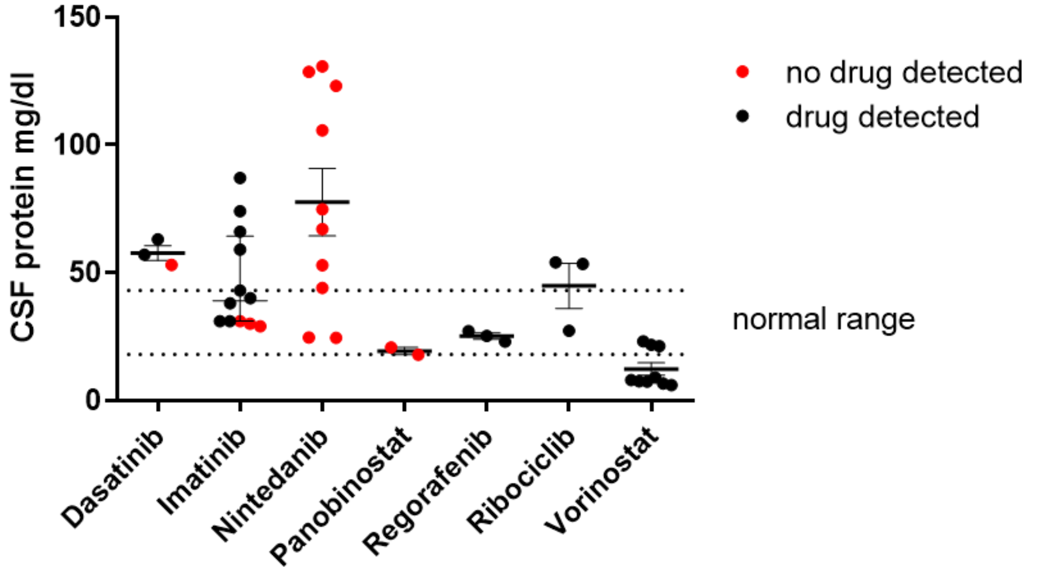

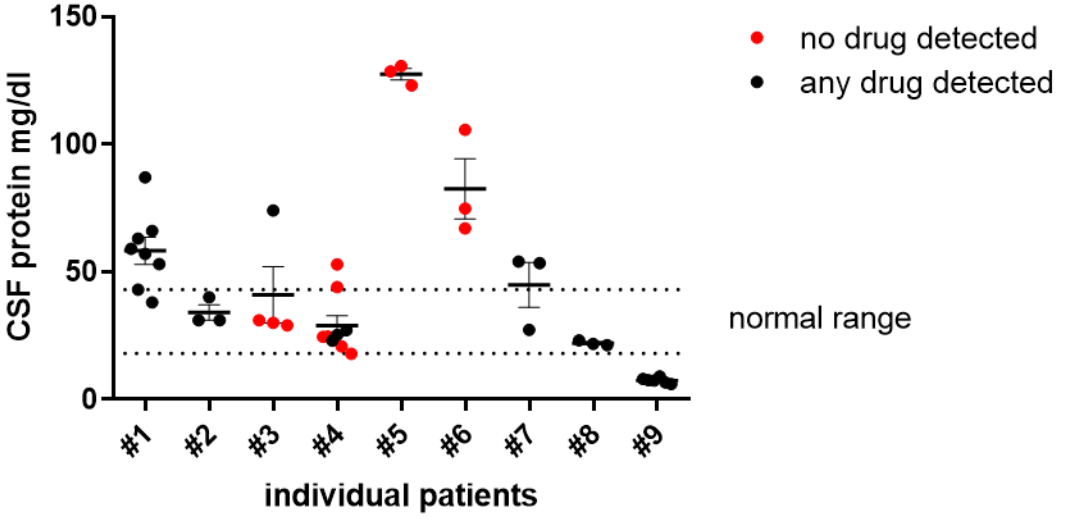

Figure S7 Matched CSF protein concentrations of all investigated CSF samples stratified for (A) drug and (B) individual patients. CSF samples where any drug was detected are indicated in black, samples without any drug detection in black.

6. <u>Experiments with ABCB1-overexpressing KBC1 cells</u>

Experiments were conducted to test for a possible inhibition or substrate character of the investigatzed drugs for ABCB1. Corresponding details are shown in Figure S8.

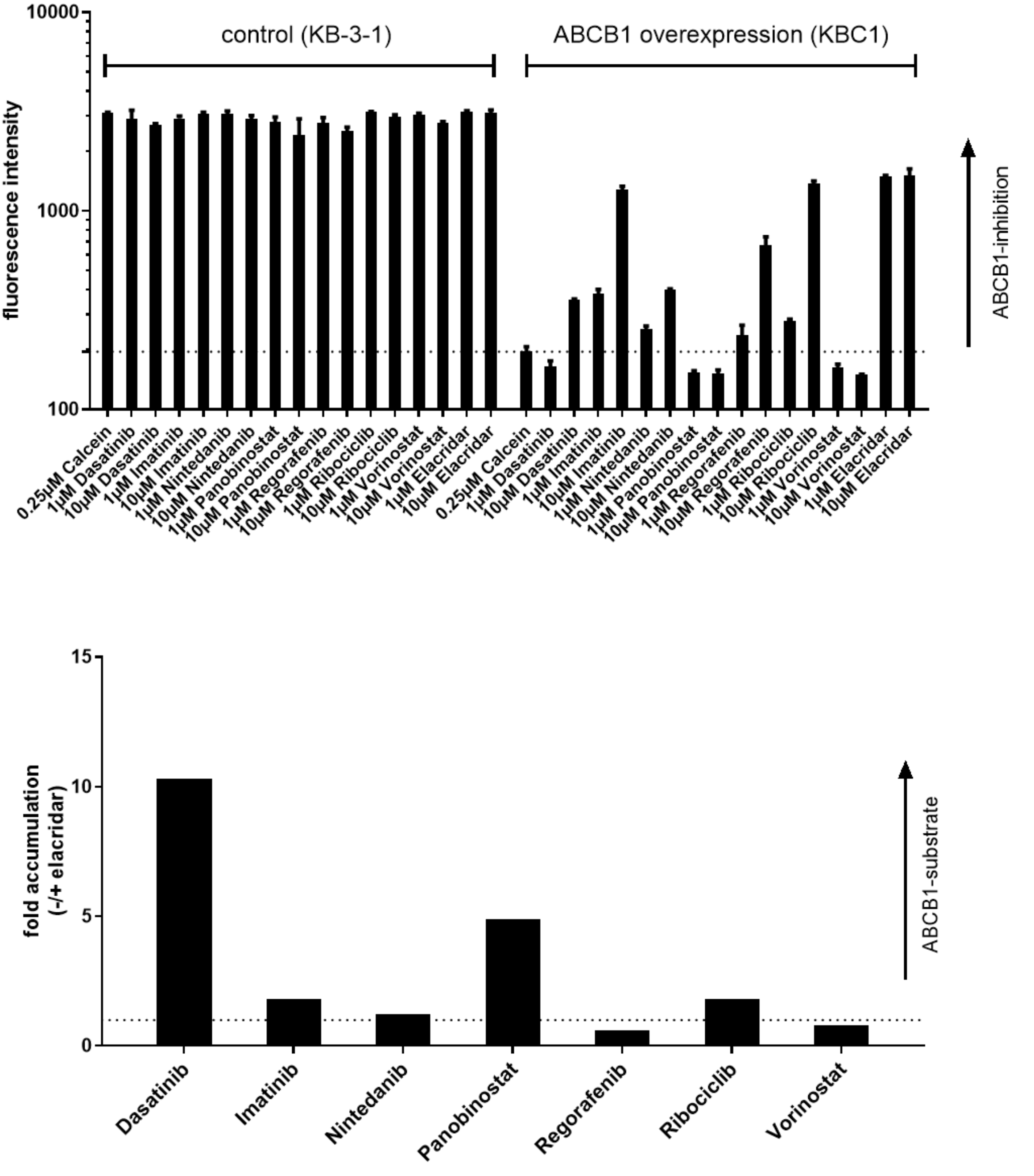

Figure S8 ABCB1 testing. (A) Calcein accumulation assay in KB-3-1 (control) and ABCB1-overexpressing KBC1 cells. Bars depict calcein fluorescence intensity measured by fluorescence activated cell sorting (FACS). Higher bars as compared to calcein in ABCB1-overexpressing cells indicate ABCB1-inhibition. Elacridar served as positive control. (B) Accumulation of drugs upon ABCB1-inhibition with elacridar measured by HPLC-QqQ MS. Results are depicted as fold-control (without elacridar inhibition).

7. Color-coding in Figure 4 of the Manuscript

Additional information on the color-coding used in Figure 4 of the manuscript is given in Table S5.

Table S5 Supplementary description of the color-coding used in the Figure 4 of the Manuscript.

	red	yellow	blue
Oral dose (mg/kg)	<1	1-5	>5
CSF concentration	not detected	< 10nM	>10nM
Molecular weight / g mol^{-1}	>500	450-500	<450
Protein binding	>99%	90-99%	<90%
Lipophilicity (S+logP)	<1,5	-	>1,5
Hydrogen bond donors (=HBD; N+O)	>5		<5
Total polar surface area (TPSA) / $Å^2$	>90		<90
Rotatable bonds	>8		<8
Collision cross section (CCS) / $Å^2$	>250	200-250	>200
ABCB1 inhibition (fold control)	>2	1-2	0-1
ABCB1 substrate	>2	1-2	0-1
logBB (ADMET Predictor)	<0	0-1	>1
BBB permeation (ADMET Predictor)	low		high
SWISS ADME	no CNS penetration		CNS penetration
